# Neurophysiological screening of individual variability for robust decoding in c-VEP-based BCI

**DOI:** 10.1162/IMAG.a.1172

**Published:** 2026-03-20

**Authors:** Sébastien Velut, Jordy Thielen, Sylvain Chevallier, Marie-Constance Corsi, Frédéric Dehais

**Affiliations:** Fédération ENAC ISAE-SUPAERO ONERA, Université de Toulouse, Toulouse, France; A&O - LISN - Université Paris-Saclay, Gif-sur-Yvettes, France; Donders Institute for Brain, Cognition and Behaviour, Radboud University, Nijmegen, Netherlands; Sorbonne Université, Institut du Cerveau - Paris Brain Institute -ICM, CNRS, Inria, Inserm, AP-HP, Hopital de la Pitié Salpetriere, Paris, France

**Keywords:** code-modulated visual evoked potential (c-VEP), electroencephalography (EEG), transfer learning, predictors, brain–computer interface (BCI), variability

## Abstract

Code-modulated visual evoked-potential (c-VEP)-based reactive brain–computer interfaces (BCIs) deliver high information-transfer rates with minimal calibration, yet performance often collapses when models are transferred between users. We, therefore, pursue a two-fold aim: first, to pinpoint neurophysiological predictors that explain this inter-participant variability; second, to identify a decoding pipeline that sustains accuracy across users in a burst-c-VEP paradigm (brief, aperiodic flashes at 3 Hz). From 24 participants, we find that stronger inter-epoch correlation (R≈0.80 ), larger peak-to-peak amplitude of the flash-VEP, larger α bandpower, larger θ bandpower, and lower δ bandpower are five neurophysiological predictors that correlate between high performers (>90% accuracy) and low performers (<70%), enabling a 22 s “go/no-go” calibration. We then compare three preprocessing schemes (small, combined, participant-specific) paired with three decoders—a convolutional neural network, a Riemannian xDAWN–LDA baseline, and GREEN, a wavelet-based symmetric positive definite neural network. Subject-specific alignment plus GREEN achieves 93% trial-level accuracy in both intra- and cross-participant settings, eliminating the 15–20% transfer loss obtained with the other tested decoding models while keeping the total calibration under 1 min. In conclusion, rapid user screening with these neurophysiological predictors, followed by this lightweight, user-specific pipeline, yields burst-c-VEP control that is fast to deploy and robust across individuals.

## 1 Introduction

A brain–computer interface (BCI) translates neural activity, typically recorded through electroencephalography (EEG), directly into machine commands. This technology facilitates communication and control without the need for muscular movement. BCIs find applications in motor function rehabilitation, immersive gaming, and the continuous monitoring of cognitive or affective states (Angrisani et al., 2021; Dehais, Ayaz, et al., 2024; Roy et al., 2022; Stampacchia et al., 2024). BCIs are usually divided into reactive paradigms—exemplified by the P300 that relies on event-related potentials (ERP) and steady-state visual evoked potentials (SSVEP)—and active paradigms such as motor imagery (MI) (Dehais, Ayaz, et al., 2024; Lotte et al., 2018). A persistent limitation of the BCI systems is the need for lengthy calibration, which can take considerable time—up to 40 min in some cases (Chen et al., 2022).

To reduce the calibration burden, many groups have shifted focus toward refining the stimulation paradigm itself (Tangermann et al., 2018). One promising approach, known as the modulated code visual evoked potential (c-VEP), involves toggling the stimulus pattern between black and white according to an aperiodic binary code—a random sequence of zeros and ones—that elicits a series of discrete visually evoked potentials (Martinez-Cagigal et al., 2021). Typically, each sequence has a near-zero cross-correlation with all other sequences, and is assigned to a specific target symbol. Two complementary decoding strategies are typically used: trial-wise classifiers, which compare an entire trial’s data to template responses (Miao et al., 2023; Thielen et al., 2021; Zarei & Mohammadzadeh Asl, 2022), and bit-wise classifiers, which reconstruct the bits in the stimulus sequences from epochs synchronized to the bits’ presentation before performing code correlation, allowing for fast and flexible decision making (Cabrera Castillos et al., 2023; Santamaría-Vázquez et al., 2025; Thielen et al., 2025; Velut et al., 2024). Due to several possible reasons—more events transmitted per unit, only one reference template easily computed if using cyclically shifted or only event responses being looked by the decoding method—calibration can be completed in under a minute (Cabrera Castillos et al., 2023; Dehais, Cabrera Castillos, et al., 2024; Thielen et al., 2021), a significant improvement over SSVEP-based systems that require training for each frequency–phase pair. Thielen et al. reduce even further the calibration time by creating a zero-calibration c-VEP BCI in an online setting, however, the BCI system still required a warm-up period of a dozens of seconds to attain the maximum performance (Thielen et al., 2021, 2025).

Despite this progress, calibration must still be repeated before each use as inter-individual factors (e.g., age, neuroanatomy) and intra-individual states (fatigue, workload, engagement) (Klimesch, 1999), compounded by environmental noise, continuously alter EEG patterns (Saha & Baumert, 2020). This recurring setup process remains a major barrier to transitioning BCIs from controlled laboratory settings to real-world applications. Contrary to other AI domains, studies in the BCI domain work with small datasets (Tangermann et al., 2018). Therefore, acquiring a large dataset to learn a foundation model is not a plausible solution. One first practical strategy to deal with user variability is to identify neurophysiological markers that forecast accuracy and then exploit those markers—either to tailor user training or to make algorithms inherently more robust. Trocellier et al. showed that, in motor-imagery based BCIs, sensorimotor-rhythm metrics, and high-gamma power are predictive of the BCI performances (Trocellier et al., 2024). Other studies tie absolute or relative power in the δ, θ, α, and β bands to fatigue, working-memory load, and attention (Klimesch, 1999; Lal & Craig, 2006; Prinzel et al., 2000; Sauseng et al., 2005). For example, an increase in the occipital α band spectrum could indicate a decrease in the focus of the participant, whereas an increase in θ band could indicate an increase in mental workload. In c-VEP paradigms, Thielen further showed that pre-task ERP latencies (N2, P2) and amplitudes (P2, N3) predicted c-VEP accuracy, whereas resting α bandpower did not (Thielen, 2025).

To complement these physiological insights, algorithmic solutions have been researched to improve the accuracy and minimize the calibration. One way would be to stay within participant and improve the decoding method. Another way would be to use information present in another participant’s data. That is, one could train the decoding model on data from other participants and fine-tune on the current participant, so-called cross-participant transfer learning. Transfer learning across participants allows to reduce the calibration time, to learn invariance to users in the data or to improve the robustness of the pipeline (Guetschel et al., 2024). Feature-space alignment techniques—ranging from Riemannian Procrustes analysis (Bleuzé et al., 2021; Khazem et al., 2021) and cyclic joint diagonalization (Altindis et al., 2023) to optimal-transport mappings (Gayraud et al., 2017; Mellot et al., 2024)—aim to bring heterogeneous participant data into a common manifold. Classifier-adaptation schemes such as leave-one-out training (Fahimi et al., 2018) and latent-alignment networks (Bakas et al., 2025; Kobler et al., 2022) refine decision boundaries online, while selective epoch-sampling and multimodal alignment further curb inter-participant drift (Jiménez-Guarneros et al., 2024; Sun et al., 2024; Xia et al., 2015). Fine-tuning foundation models have proved to be especially effective: freezing most layers and adapting only the task-specific heads can raise participant-adaptive accuracy with minimal additional data (Aristimunha et al., 2023; Guney et al., 2022; Junqueira et al., 2024; Liu et al., 2018). Conversely, unfreezing early convolutional layers as Ding et al. (2025) demonstrated further boosts in cross-participant performance for SSVEP paradigms even when only a subset of stimulus classes is available for calibration. Most recently, promising approaches leverage the ability of wavelet representations to capture both temporal and spectral structure in EEG with the efficiency of deep learning search space. Those wavelet-based deep networks (Naik-M & Ahamed, 2024; Paillard et al., 2025) define lightweight yet accurate classifiers, thanks to their rich latent representational space.

Most existing solutions remain too computationally heavy or paradigm bound for real-time use. Bridging laboratory precision with field-ready practicality, therefore, calls for a unified approach that marries neurophysiological predictors for user-centered adaptation with transfer-learning pipelines capable of rapid, generalizable decoding. We hypothesize that investigating the neurophysiological patterns of visual evoked responses would allow for the identification of markers indicating inter- and intra-individual differences in performance. Moreover, these neurophysiological predictors could facilitate the development of a more efficient transfer learning pipeline, thereby reducing calibration time. To validate these hypotheses, we used a dry-EEG burst-c-VEP dataset previously collected by Dehais et al. (Dehais, Cabrera Castillos, et al., 2024) from 24 healthy participants. We first revisit whether spectral band power, ERP peak-to-peak amplitude, ERP latency, and intra-trial correlation explain residual inter-user differences in accuracy. We then test whether participant-specific Riemannian alignment coupled with lightweight deep learners—the GREEN architecture (Paillard et al., 2025), an EEG2Code-inspired convolutional neural networks (CNN) (Cabrera Castillos et al., 2023), and a tangent-space LDA pipeline (Rivet et al., 2009)—can achieve, with a fine-tuning on the current participant, light-calibration cross-participant decoding. Three preprocessing regimes (small, combined, participant-specific) are benchmarked, and we quantify both the resulting accuracy gains and the variance captured by the spectral and ERP predictors.

## 2 Methods

### 2.1 Dataset description

All the results presented in this study are based on an already collected dataset called Ricker patch-based StAR c-VEP (ISAE-SUPAERO Dataset (Cabrera Castillos & Dehais, 2024)). The research received approval from the ethics committee of the University of Toulouse (CER approval number 2023-749) and was conducted in compliance with the Declaration of Helsinki. The EEG data were collected using a dry 8-electrode Enobio system, with a sampling rate of 500 Hz to capture the surface brain activity. The eight electrodes were positioned over the occipital and parieto-occipital sites, namely PO7, O1, Oz, O2, PO8, PO3, POz, PO4. This dataset comprised 24 healthy individuals (4 women, 20 men, mean age = 29.3 years, SD = 7.5 years) performing a visual task where they were instructed to direct their attention sequentially to 5 presented targets for 2.2 s each, which were cued in a random order for 0.5 s each. Each participant performed 15 runs, each of which were composed of 5 trials of 2.2 s each. In other words, we had a total of 75 trials including 15 trials of 2.2 s per class (N = 5).

### 2.2 Preprocessing

**Fig. 1.**
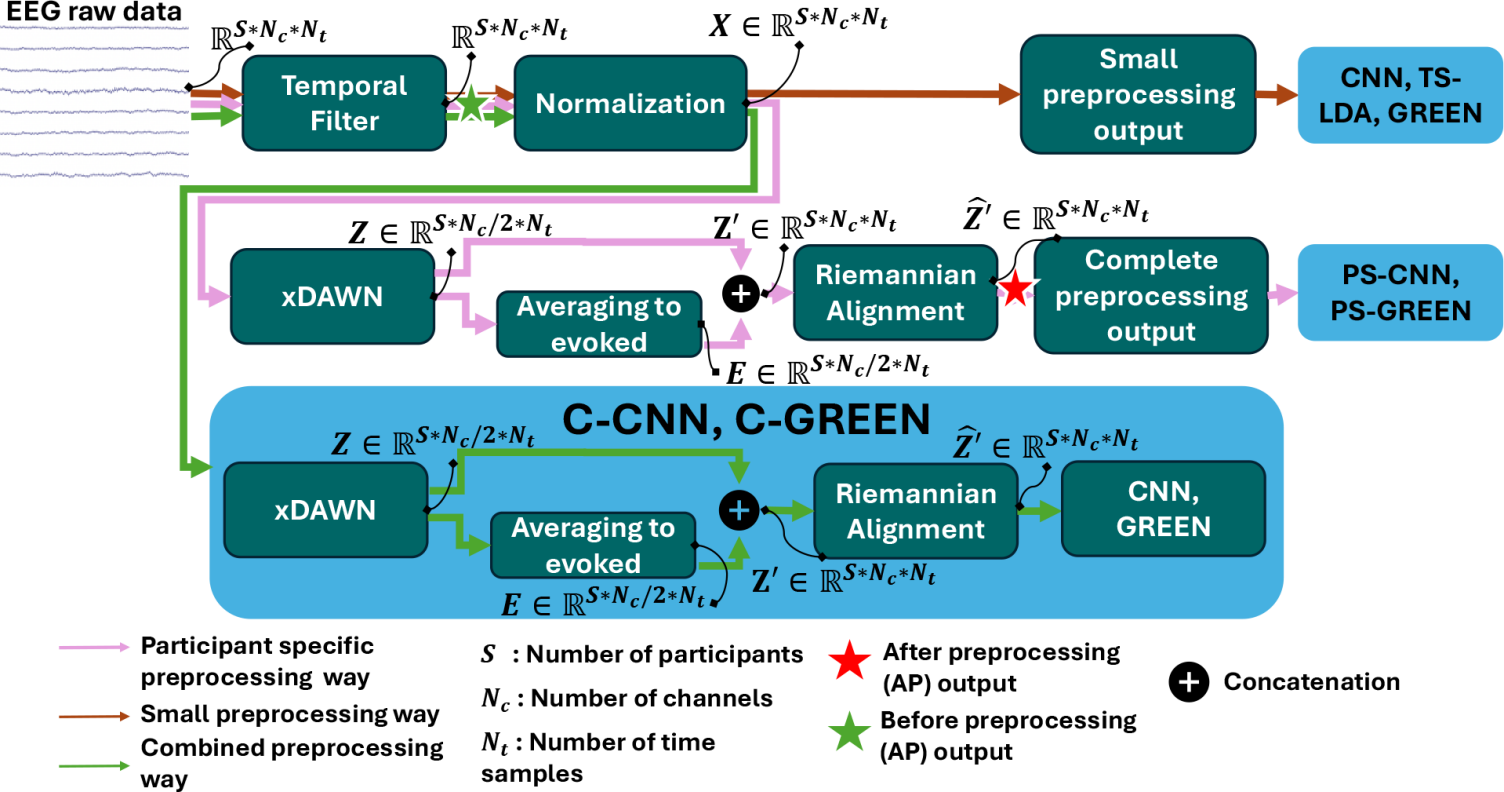
Schematic visualization of the steps involved in the preprocessing workflow. Output data dimension are indicated after each step. The model used at the end of each preprocessing way is shown in light blue squares.

The preprocessing was not exactly the same for all the decoding pipelines, see Figure 1 for a summary. First, for all decoding pipelines, to restrict to the frequency bands involved during c-VEP neural processes, we applied a finite impulse response filter between 1 Hz and 45 Hz. As the time between two onsets of the flashes in a code is at least bigger than 0.35 s, we created an overlapping epoch of size 0.35 s every 1/500 s. This gives $N_{e}=15*\left(2.2-0.35\right)*500=13875$ epochs per participant per class. We chose to create epochs of 0.35 s every 1/500 s rather than epochs of 0.35 s every 1/60 s (i.e., one epoch per video frame) because the latter configuration did not provide a sufficient number of training samples for the deep learning classifier. The denser temporal sampling was, therefore, required to ensure stable classifier training. Then, to ensure comparable amplitude across participants, we normalized the data by dividing it by the standard deviation of each participant’s training set. This marks the conclusion of the small preprocessing scheme.

To improve the quality of the signal and reduce the variability between participants, we introduced a more complete preprocessing. To improve the signal-to-noise ratio (SNR) of the captured ERP response to the flashes of the StAR c-VEP, the preprocessing consisted of a spatial filtering using xDAWN, which enhances performance for ERP-based BCI (Rivet et al., 2009). From xDAWN, we selected the four highest ranked components. The number of components chosen by xDAWN was fine-tuned and four components showed the highest performance. The fine-tuning was performed on the training set by evaluating epoch-level accuracy for xDAWN configurations ranging from two to eight components, with a step size of one, and selecting the configuration that yielded the highest epoch-level accuracy. xDAWN is a spatial filter designed for ERP-based BCI and we had ERP responses after each flash of the StAR c-VEP code. xDAWN starts by decomposing the signal into two parts, one part corresponding to the noise and the on-going activity of the user brain, one part corresponding to the multiplication of the ERP signal matrix and a Toeplitz matrix. With some transformation over the terms by applying singular values decomposition and QR factorization, this spatial filter is described as

$$\text{X}=\text{D}{\text{A}}^{\prime }\;{\text{W}}^{⊤}+{\text{N}}^{\prime },$$

where D is the Toeplitz matrix, ${\text{A}}^{\prime }$ is the synchronous response of reduced dimensions, $\text{W}={\text{R}}_{\text{X}}^{⊤}\Psi_{\text{s}}$ is its spatial distribution over sensors, with ${\text{R}}_{\text{X}}^{⊤}$ an upper triangle matrix and $\Psi_{\text{s}}$ an orthogonal matrix, and ${\text{N}}^{\prime }$ corresponds to the noise term. Finally, the enhanced signal Z is obtained by multiplying the signal X with the spatial filter $\text{Û}={\text{R}}_{\text{X}}^{-1}\Psi_{\text{s}}$, that is, Z=XÛ .

We create super epochs by combining the evoked responses of the spatially filtered epoch with each spatially filtered target response, see Figure 1. Let ${\text{E}}_{\text{Z}}=\frac{1}{N_{e}}\sum_{k=1}^{N_{e}}{\text{Z}}_{\text{k}}$ be the evoked response of the spatially filtered data, where $N_{e}$ is the number of epochs and ${\text{Z}}_{\text{k}}$ is the $k^{th}$ epoch of the spatially filtered target data. We create the super epochs where the $k^{th}$ sample will be $Z_{k}^{\prime }=\{Z_{k,1},Z_{k,2},Z_{k,3},E_{\hat{Ζ},1},E_{\hat{Ζ},2},E_{\hat{Ζ},3},E_{\hat{Ζ},4}\}$. The concatenated evoked response allows preservation of the information contained in the average target response. By combining this preserved average response across epochs with the application of the spatial filter, intra-epoch variability is mitigated. The evoked response of the spatially filtered data is computed using the calibration data and is then concatenated to all epochs, including both the training and test sets.

To align the different participants on the same feature space and reduce the difference between the data of the participants, we applied a Riemannian spatial alignment, as shown in Figure 1. For this purpose, we are, first, using the calibration phase of the participant as a training batch and computing the associated covariance matrices with the Ledoit–Wolf estimator (Ledoit & Wolf, 2004). In a second time, we are measuring the Riemannian mean of these matrices, and finally, we are transporting the samples of the participant with this mean as follows:

$$\hat{\text{Z}}\prime_{i}=\bar{\text{R}}\times {{\text{Z}}^{\prime }}_{\text{i}}\;\forall i\in \left[1,\ldots ,N_{e}\right],$$ (1)

where $Z_{i}^{\prime }\in {\mathbb{R}}^{N_{c}\times N_{t}}$ is an element of ${\left\{{{\text{Z}}^{\prime }}_{\text{i}}\right\}}_{i\in \left[1,....,N_{e}\right]}$ and contains the different xDAWN filtered epochs of a participant, $\bar{\text{R}}$ is the Riemannian mean of the set $\left\{Z_{1}^{\prime },...,Z_{T}^{\prime }\right\}$ and T is the number of samples in the calibration set of the participant.

Overall, the data of the test dataset were never used for hyperparameter optimization, visual inspection of the data, computation of the neuro-predictors, or any training of the preprocessing steps. All decisions were made exclusively using the calibration/training data, ensuring that no test information influenced the reported results.

### 2.3 Neurophysiological predictors

To investigate the differences between participants and identify potential predictors of performance, we pre-selected several features from the target epochs. The measurements were performed at four different steps: (i) before preprocessing (BP), that is from data only filtered between 1 Hz and 45 Hz; (ii) after complete preprocessing (AP), that is from data transformed with all of the complete preprocessing steps; (iii) before preprocessing and transformed in the wavelet space (BPWave), meaning a wavelet transformation is applied on the temporarily filtered data; (iv) after complete preprocessing and transformed in the wavelet space (APWave).

We visually inspected all channels in BP and xDAWN components extracted in AP to confirm the workflow correctness by checking presence of artifact, noise, and outliers. These visual checks have been performed on the training set to avoid data leakage. In all but one case, the channels in BP and xDAWN components in AP were validated. For 1 participant out of 24 (participant 2), the first xDAWN component was null as it captured mostly noise due to a particularly low signal-to-noise ratio (SNR). In this sole case, we removed the first component and shifted the components such that the second component became the first, the third became the second, and so on.

Five potential neurophysiological predictors were computed, namely the peak-to-peak amplitude, the time of the maximal peak, the power spectra, the SNR, and the correlation between target epochs.

#### 2.3.1 Peak-to-peak amplitude

To measure the peak-to-peak amplitude, we averaged the target epochs. In the BP and BPWave spaces, we calculated the difference between the maximum and minimum values in Oz channel, as Oz channel showed the largest peak-to-peak amplitude. In the AP and APWave spaces, we computed the difference between the maximum and minimum values in the first xDAWN component, which accounts for the largest peak-to-peak amplitude of the signal.

#### 2.3.2 Latency of the strongest peak

To determine the time of the strongest peak, we took the absolute value of the average target epoch to account for cases where the strongest peak was negative or positive. We determine the time associated with the maximum absolute value. As the peak-to-peak amplitude, for BP and BPWave spaces, we took the data in the Oz channel while we took the data in the first xDAWN component for AP and APWave spaces.

#### 2.3.3 Power spectral density

To obtain the power spectral density (PSD), we applied the Welch method using the MNE library (Gramfort et al., 2013) across different frequency bands: the δ band (1–4 Hz), the θ band (4–8 Hz), the α band (8–13 Hz), and the β band (13–35 Hz). Then, to ensure comparable PSD amplitude across participants, the PSD values were divided by the mean of the PSD of the data calculated between 1 Hz and 45 Hz. In BP, we considered the eight channels available, whereas in the AP space, we considered only the first four xDAWN components before calculating the general PSD and the PSD of the sub-bands as the last four xDAWN components correspond to the average of all target epochs. After calculation of the PSD with the Welch method, all these channels were averaged. As the interpretable information about frequency is lost here when going in the wavelet space, the power spectral density was not measured in the BPWave and APWave spaces.

#### 2.3.4 Signal-to-noise ratio

To measure the SNR, we computed the average PSD of target epochs for each participant between 1 Hz and 45 Hz and divided it by the mean of the PSD of non-target epochs. In BP, we considered the eight channels available, whereas in the AP space, we considered only the first four xDAWN components before calculating the general PSD and the PSD of the sub-bands as the last four xDAWN components correspond to the average of all target epochs. After calculation of the PSD with the Welch method, all these channels were averaged. As the interpretable information about frequency is lost here when going in the wavelet space, the SNR was not measured in the BPWave and APWave spaces.

#### 2.3.5 Correlations between target epochs

To measure the correlation between each target epoch, the data in the BP and BPWave spaces were reduced to the Oz channel, and the data in the AP and APWave spaces were reduced to the first component. We calculated the Spearman correlation, which is less sensitive to outliers or peaks in the temporal data than Pearson correlation, between all epochs to obtain a correlation matrix for each participant, which is of size epochs x epochs. To avoid the effect of the auto-correlation of the epochs in the final measure, we reduced the correlation values to the upper-diagonal entries in the correlation matrices. Subsequently, we measured the mean of all upper-diagonal correlation values for a given participant. Higher values indicated lower variability between target epochs. We focused solely on the target epochs to emphasize the stability of the response to a flash and to minimize the effect of variance and noise, which are prominent for non-targets, that is, no external stimulus.

### 2.4 Decoding models

In this paper, we considered three decoding models, namely (i) a variant of the convolutional neural network EEG2Code (Nagel & Spüler, 2019) that has been successful in c-VEP classification (Cabrera Castillos et al., 2023) called here CNN; (ii) a Riemannian model that has been used in P300-based BCI and used for the online setup in Dehais, Cabrera Castillos, et al. (2024), referred to as tangent space linear discriminant analysis (TS-LDA) (Barachant et al., 2012); (iii) a wavelet-based deep learning model called Gabor Riemannian EEGNet (GREEN) (Paillard et al., 2025).

#### 2.4.1 CNN

We used a variant of the original EEG2Code model (Nagel & Spüler, 2019) that was later optimized for burst-c-VEP decoding (Cabrera Castillos et al., 2023). The model is structured with three convolutional blocks. The first one is a spatial convolution block, including one Conv2D layer with one kernel of size ($N_{c}$, 1), a batch norm layer, a max pooling layer, and a dropout layer with a dropout rate of 0.5. The next two blocks are one temporal convolution and one spatio-temporal convolution, which include a leaky ReLU added before the max pooling layer and a dropout layer after the max pooling layer with a dropout rate of 0.5. These two Conv2D layers have one kernel each of size (1, 32) and (5, 5) and a dilatation rate of (1,2) and (2,2), respectively. To finish, a 256-unit dense layer with a leaky ReLU activation function followed by a 2-unit dense output layer with a softmax activation function, together predict the occurrence of flashes. The CNN model was compiled with an Adam optimizer with a learning rate of 0.001.

#### 2.4.2 TS-LDA

This model is a Riemannian machine learning algorithm that is faster in compute time, has a smaller number of parameters than the CNN (1050 parameters for TS-LDA vs 28353 parameters for CNN), and it is used in P300-based BCI (Rivet et al., 2009). This model could be resumed as a spatial filter combined with a classifier that uses second-order statistics, that is the covariance information. The first layer spatially filters the EEG data with the xDAWN filter by choosing, in our case, the first four components and is fitted only on epochs with the label 1. Covariance estimated with Ledoit–Wolf (Ledoit & Wolf, 2004) for each of the four components are concatenated with the target signal covariance to create a super-covariance matrix. To be able to predict the label, the covariance matrices of the selected xDAWN components are projected to the tangent space where a linear discriminant analysis (LDA) classifier is applied on the tangent vectors.

#### 2.4.3 GREEN

GREEN is an algorithm introduced in Paillard et al. (2025) to detect novel neuro-markers applicable in a medical context. To get temporal and frequency information from the EEG data and to increase the quantity of usable information, GREEN starts by performing a Gabor wavelets transform, followed by a 1D convolutional layer.

In this paper, we choose to separate the signal in 22 frequency bands that are learned through training, all included between 0 and 4.4 octaves. Then the wavelets data are transformed in covariances matrices and go through an SPDNet (Huang & Van Gool, 2016) of one block including of a shrinkage layer, a BiMap layer, and a ReEig layer. The shrinkage layer ensures that the covariance matrices are positive definite, the BiMap layer is reducing the dimension, and the ReEig layer is a regularization layer to avoid getting null eigenvalues. We parametrize the SPDNet outputs to four channels, to ensure reliable performance and a comparable latent space with TS-LDA. A fully connected layer with 2 hidden layers of size 20 and 10 units is processing the tangent space projection of the latent space to make classification decisions. The hyperparameters of this algorithm—number of main frequencies in the wavelet family (6 to 30 with 2-step increment), dropout rate for fully connected layers (0.4 to 0.8 with 0.1-step increment), and maximal center frequencies of the wavelets in octaves (3.6 to 5.0 with 0.2-step increment)—were optimized with a 5-fold grid search cross-validation training scheme.

#### 2.4.4 Decoding procedure

In the burst-c-VEP paradigm, we decode which code the participant is looking at on the trial level (N = 5), by first classifying whether the participant is looking at a flash or not at the epoch level (N = 2, with labels 1 or 0, respectively).

With the reconstructed series of 0’s and 1’s, we used Pearson’s correlation to compare it with the codes flashed on the screen and return the code index that resulted in the maximum correlation. This correlation maximization is performed at each epoch using all previous epochs, and we use a dynamic stopping rule to determine the final index of the code. Specifically, we emit the trial level decoding if the last 30 predictions resulted in the same code index, otherwise the decoding continues with the next epoch.

There are approximately four times more 0’s than 1’s, due to the nature of the StAR c-VEP codes. We re-balanced our training set between the number of 0’s and 1’s using a random undersampler. The testing set was not balanced to simulate an online context as closely as possible. We evaluated alternative class-imbalance handling strategies, such as oversampling, during a related study (Velut et al., 2024). These approaches did not yield promising improvements compared with undersampling, and we, therefore, retained the undersampling strategy in the present work.

### 2.5 Evaluation of the preprocessing schemes

Three preprocessing schemes are evaluated for the CNN and the GREEN algorithms. The first one uses only the small preprocessing steps and the algorithms are called GREEN and CNN.

In the second setting, the complete preprocessing steps are included in a pipeline within the model and are, therefore, fitted with the model by using the training set. We call this a combined scheme, hence denoted the algorithms C-GREEN and C-CNN.

In the last setting, for each participant, the complete preprocessing steps are fitted with a calibration set but all the data are transformed with the fitted complete preprocessing steps before training the model. This last one corresponds to a participant-specific preprocessing. We call this scheme participant specific (PS) and the associated algorithms are named PS-GREEN and PS-CNN.

For the TS-LDA, the spatial filtering with xDAWN is already included in the algorithm through the xDAWN filter. Thus, there is no need to investigate different preprocessing steps. Moreover, the xDAWN parameters in TS-LDA are estimated per training fold.

In summary, we benchmarked seven models: *TS-LDA*, *CNN*, *C-CNN*, *PS-CNN*, *GREEN*, *C-GREEN*, *PS-GREEN*.

### 2.6 Training procedure

**Fig. 2.**
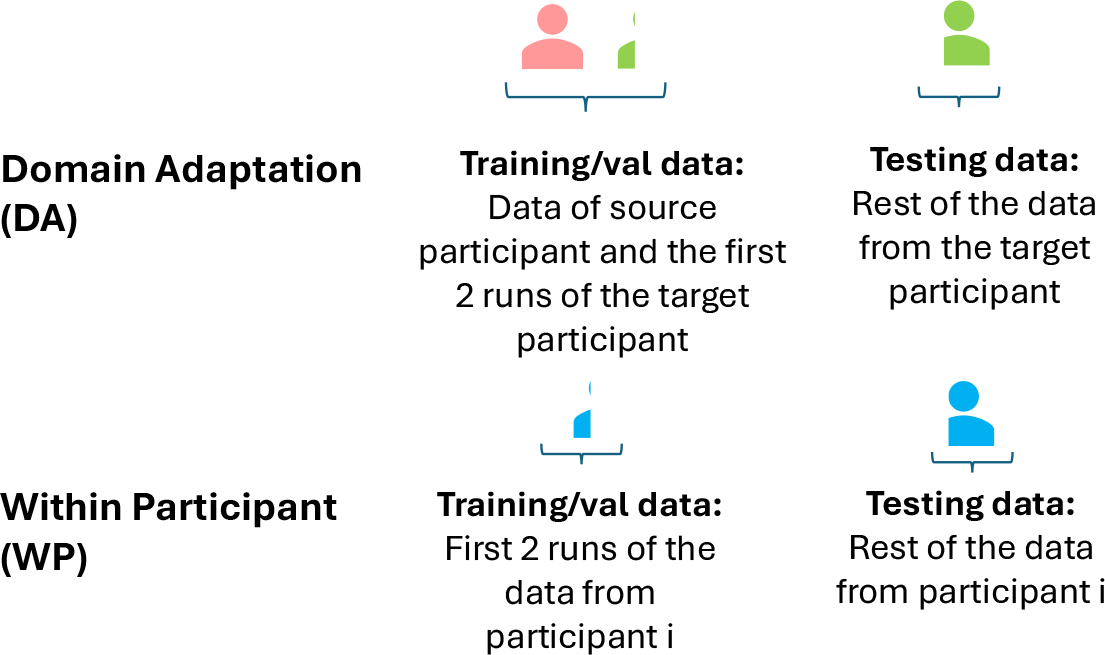
Graphical explanation of domain adaptation (DA) and within participant (WP) training procedures.

We investigated two training procedures (see Fig. 2). The first one, referred to as Within Participant (WP) procedure, corresponded to a classic train/test method where the data of a participant i are split into a training set and a testing set. The training set used 2 out of the 15 total runs.

The second training procedure corresponded to a Domain Adaptation (DA) procedure. The training set corresponded to the concatenation of the data of one source participant s and the first two runs of the target participant t. The rest of the data of the target participant was used in the testing set.

More specifically, if $Z_{s}\;:\left\{Z_{s1},...,Z_{s15}\right\}$ refers to the data of the source participant s, where $Z_{sj}$ are the samples of the $j^{th}$ trial of the source participant s and $Z_{t}\text{​}:\left\{Z_{t1},...,Z_{t15}\right\}$ the data of the target participant t, where $Z_{tj}$ are the samples of the $j^{th}$ trial of the target participant t. Then in DA, the training set will be $\Omega_{train}=Z_{s}\cup Z_{t1}\cup Z_{t2}$ and the testing set will be $\Omega_{test}=Z_{t}\backslash \left\{Z_{t1},Z_{t2}\right\}$.

For both WP and DA procedures, the total amount of calibration data from the target participant is 2×5×2.2=22 s, while for DA an additional x seconds of data are used from other source participants.

For both the WP and DA procedures, all 24 participants acted as a target participant once.

Note that for the DA procedure, we also iterated through all 23 participants other than the target one to be used as source to be trained on. In this manner, for DA, each participant was used once as a target participant and 23 times as a source participant.

### 2.7 Statistical analyses

For the linear correlation between the neurophysiological predictors and the performance, an ordinary least squares (OLS) linear regression was used to find the linear curve. The associated p-value was found using a Wald test.

For the analyses of the classification accuracy of each model, the statistical differences were obtained using the Stouffer’s method that combines p-values resulting from the Wilcoxon signed rank test. The effect size between two decoding models is measured via standardized mean difference (SMD) estimated over the participants. A one-way repeated measure ANOVA, with factors “base model” (CNN, LDA, GREEN) and “preprocessing type” (small, combined, and participant-specific), was computed for each objective metric (epoch-level accuracy, trial-level accuracy, training compute time, prediction compute time of the test set, epoch-level recall, and epoch-level f1 score). The tests were two sided and completed with Bonferroni correction. Significance level was set at p<0.05 for all analyses.

## 3 Results

### 3.1 Neurophysiological predictors

#### 3.1.1 Spectral neurophysiological predictors

**Fig. 3.**
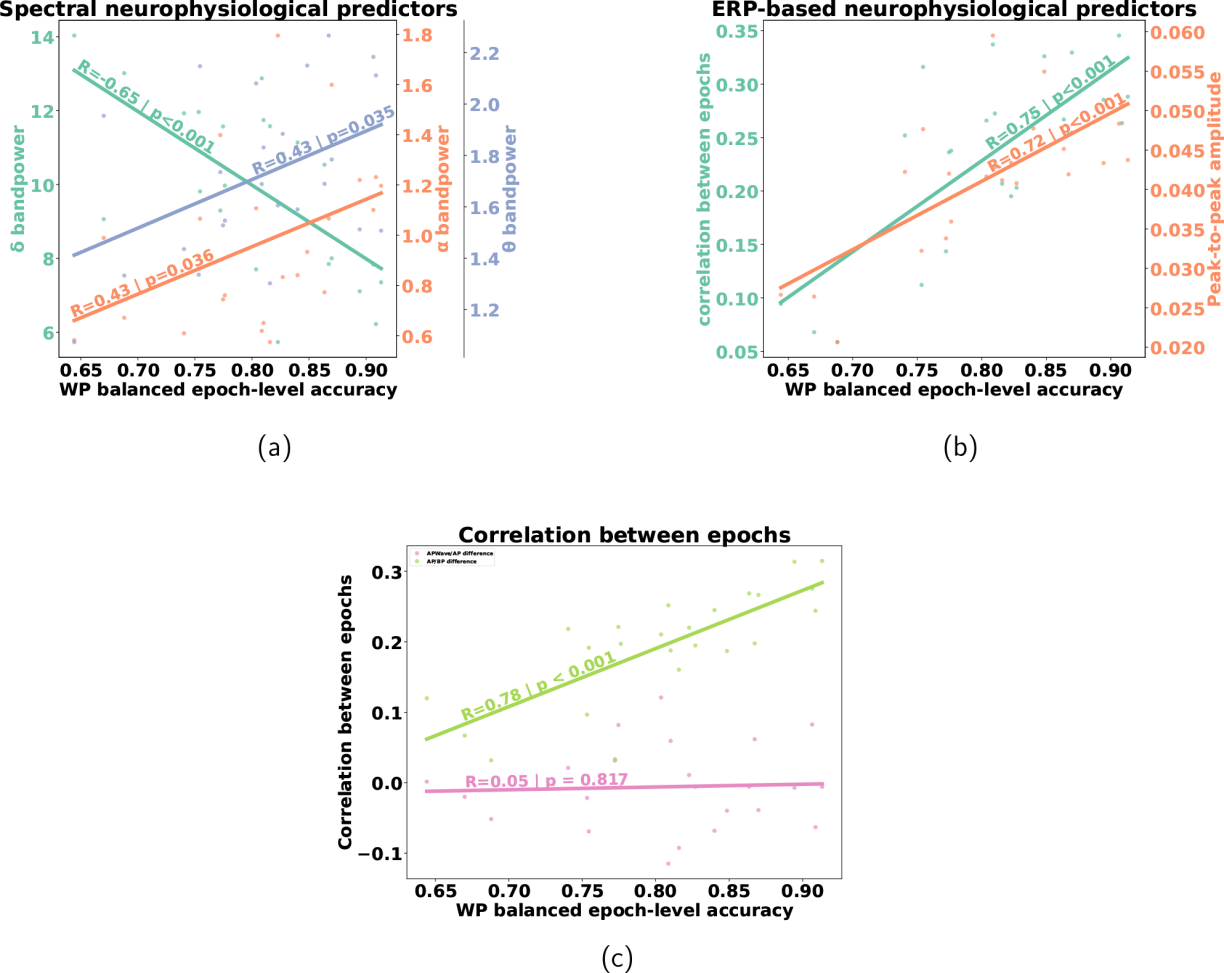
Linear correlation between the balanced epoch-level accuracy in WP training procedure and (a) the spectral neurophysiological predictors (δ, θ, and α bandpower). (b) The ERP-based neurophysiological predictors (correlation between epochs and peak-to-peak amplitude). (c) The difference in inter-epochs correlation measured in two different neurophysiological predictors measurement space (APWave/AP and AP/BP). The *p*-value and the Pearson correlation coefficient are written above the lines.

The Wald test revealed a statistically significant linear correlation between the PSD in the α bandpower (R=0.43 and p=0.036 ), the θ bandpower (R=0.43 and p=0.035 ), and the δ bandpower (R=−0.65 and p<0.001 ), computed in AP space, and the PS-GREEN balanced epoch-level accuracy in the WP procedure (see Fig. 3a), or in the DA procedure (see Supplementary Fig. SF4). The correlation of the δ bandpower was stronger and inversely proportional to the balanced epoch-level accuracy, whereas the correlation of the α and θ bandpower was smaller and proportional to the balanced epoch-level accuracy. No significant correlations were found for the β bandpower (R=0.38 and p=0.066 ).

#### 3.1.2 ERP-based neurophysiological predictors

The Wald test revealed a linear correlation between the PS-GREEN balanced epoch-level accuracy in WP and two ERP-based features: the peak-to-peak amplitude (R=0.84 and p<0.001 ) and the correlation between target epochs (R=0.72 and p<0.001 ), both computed after the participant-specific preprocessing (see Fig. 3b).

The inter-target epoch correlation was slightly more correlated than peak-to-peak amplitude, though both were positively correlated with balanced epoch-level accuracy. No significance was found for the strongest peak latency (R=−0.39 and p=0.056 ) nor for the SNR (R=0.11 and p=0.609 ).

#### 3.1.3 Impact of the neurophysiological predictors measurement space

To assess whether preprocessing or wavelet transformation directly affected the correlation between balanced epoch-level accuracy and neurophysiological predictors, we computed the correlation between balanced epoch-level accuracy and the differences of the neuro-marker values computed across conditions: between AP and BP, and between AP and APWave.

OLS regression and the Wald test revealed a significant correlation between the difference in values between AP and BP and decoding performance (p<0.001 ), suggesting a meaningful effect of measurement space (see Fig. 3c). Specifically, neurophysiological predictors computed in AP space provided better predictions of performance than the ones computed in BP space. No significant difference was found between AP and APWave (p=0.498 ).

### 3.2 Aggregate decoding performance

To measure the performance of a model, we calculated the balanced epoch-level accuracy for decoding the label 1 or 0 of each frame, the trial-level accuracy, the epoch-level recall score, the epoch-level f1 score, the training compute time of the model (do not include the preprocessing time), and the prediction compute time.

The best model in the WP procedure for the epoch-level and trial-level accuracy is C-CNN reaching 0.83 (0.93 for trial-level accuracy), while CNN, PS-CNN, and PS-GREEN are closely behind with a 0.03, 0.01, and 0.02 (0.05, 0.01, and 0.01) lower score, respectively. The Stouffer’s method disclosed that C-CNN and PS-CNN performed significantly better than the five other decoding models (p<0.01 ). In the DA procedure, the Stouffer’s method disclosed that the best model is PS-GREEN reaching 0.81 and 0.93, (p<0.001 ). PS-GREEN is the only model that maintains the same accuracy between the WP procedure and the DA procedure, showing that it succeeded in countering the inter-participant variability. Moreover, PS-GREEN has the smallest range between the min and max accuracy in DA, meaning the score seems consistent across participants (see Table 1, a detailed version of the balanced epoch-level accuracy for each decoding pipeline and each subject is present in Supplementary Table ST1).

**Table 1.** Epoch-level and trial-level accuracy and their standard deviation for the domain adaptation (DA) and within participant (WP) training procedure.

	DA		WP	
Method	Epoch-level accuracy	Trial-level accuracy	Epoch-level accuracy	Trial-level accuracy
C-GREEN	0.72±0.08	0.76±0.19	0.80±0.07	0.68±0.15
GREEN	0.55±0.05	0.34±0.13	0.54±0.03	0.28±0.09
PS-GREEN	**0.81**±**0.06**	**0.93**±**0.08**	0.81±0.07	0.92±0.09
TS-LDA	0.66±0.09	0.62±0.22	0.79±0.08	0.90±0.10
C-CNN	0.71±0.09	0.72±0.20	**0.83**±**0.06**	**0.93**±**0.09**
CNN	0.73±0.09	0.76±0.20	0.80±0.07	0.88±0.15
PS-CNN	0.77±0.07	0.84±0.15	0.82±0.06	0.92±0.09

In bold, we have the best results per columns. For DA, the accuracies are calculated across the S*(S-1) calculated scores.

**Fig. 4.**
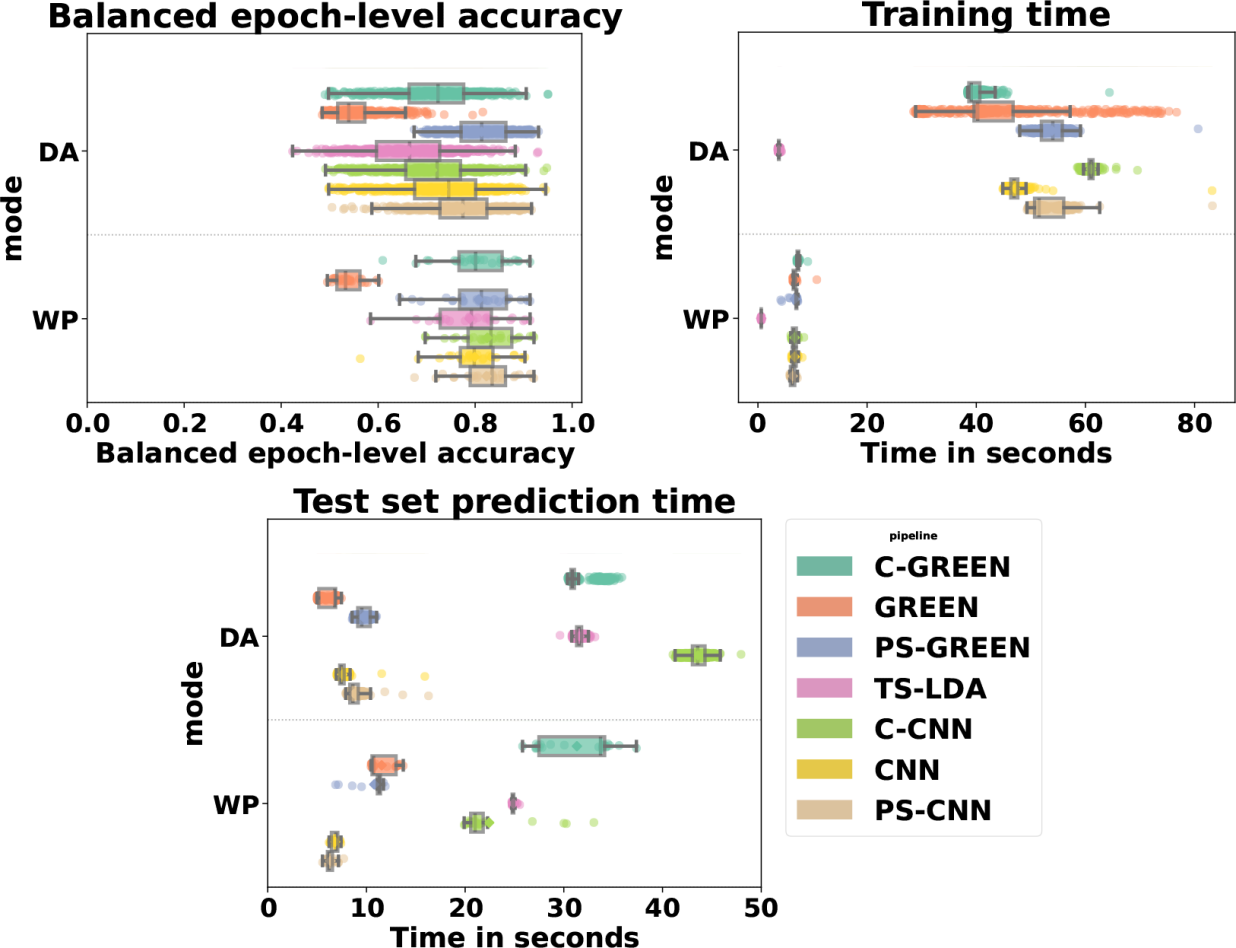
Balanced epoch-level accuracy (top left), total training compute time (top right), and total prediction compute time (bottom middle) for the seven methods C-GREEN, GREEN, PS-GREEN, TS-LDA, C-CNN, CNN, PS-CNN. Up: DA procedure, Down: WP procedure. The boxes correspond to the inter-quartile range, the bar in the boxes to the median, the bold square to the mean.

For the training compute time, the Stouffer’s method disclosed that TS-LDA has the smallest training compute time in WP and DA procedures, reaching 3.68 s in DA and 0.61 s in WP. In WP, C-GREEN achieved 7.41 s for the computed time for the training and has a significantly higher training compute time than the other models (p<0.001 ). In the DA procedure, C-CNN has the highest training compute time, followed by PS-GREEN and PS-CNN, reaching, respectively, 58.75, 51.93, and 51.03 s (see Fig. 4).

For the prediction compute time across all test trials, the Stouffer’s method disclosed that TS-LDA and C-CNN have the highest prediction compute time in WP and DA procedures going up to 30.25 and 42.71 s in DA and 24.90 and 22.44 s in WP. The three lowest prediction compute times in WP and DA are attained with CNN, PS-CNN, and PS-GREEN reaching 7.24, 8.67, and 9.73 s in DA and 6.83, 6.36, and 10.79 s in WP (see Fig. 4).

Upon examining the performance details per participant, it is evident that some participants consistently achieved balanced epoch-level accuracy under 70% regardless of the source participant chosen, see Supplementary Figure SF2. While some of these performance issues could be explained by the neurophysiological predictors described in Section 3.1.1, which exhibit a linear correlation with performance, other measures might account for the lower performance observed in certain participants. For example, for Participant 19, the peak latency neurophysiological predictor is significantly smaller (p<0.001 ) than that of the other 23 participants (0.10 s compared with 0.20 s), potentially explaining the lower performance. Another example is Participant 2, who has a low SNR, which could explain the reduced performance for this participant.

A graph presenting, similar to Figure 4, the distribution of all measures, meaning with trial-level/recall and f1 score in addition, is present in the Supplementary files, see Supplementary Figure SF1.

### 3.3 Effects of preprocessing

The one-way repeated measures ANOVA disclosed a main effect of the preprocessing on the epoch-level accuracy ($F_{2,46}=313.73$, p<0.001 ). Post hoc analyses indicated that the participant-specific preprocessing was significantly better (p<0.001 ) in DA procedure than in both the combined one and the small preprocessing. With participant-specific preprocessing, the trial-level accuracy improved by 15% in average and the epoch-level accuracy improved by 7.5% in average in DA compared with the combined one.

The combined preprocessing is significantly better (p<0.001 ) than the small preprocessing in the WP procedure and only for the GREEN-based model in the DA procedure. No significant differences (p=0.57 ) were found in WP for the CNN-based model between participant-specific and combined preprocessing (see Fig. 4, Table 1).

### 3.4 Effects of model choice

The one-way repeated measures ANOVA disclosed a main effect of the base model on the epoch-level accuracy ($F_{2,46}=169.91,p<0.001$ ). Post hoc analysis revealed that CNN-based models have better epoch-level accuracy than GREEN-based model and TS-LDA in WP procedure (p<0.001 ). In the DA procedure, the CNN-based model performed better than the TS-LDA and GREEN-based models, although PS-GREEN remained the top-performing model.

GREEN and PS-GREEN models, contrary to CNN-based model and TS-LDA, maintained a consistent balanced epoch-level accuracy across the DA and WP procedure, revealing the strength of the GREEN-based models to counter the inter-participant variability (see Fig. 4, Table 1).

The two-way repeated measures ANOVA disclosed a main effect of the pair base model/preprocessing on the epoch-level accuracy ($F_{2,46}=572.33,p<0.001$ ).

### 3.5 Effects of pretraining

We evaluated a pre-training strategy in which one classifier was first pre-trained for each source participant and then fine-tuned on the target participant. The effect of this approach is illustrated in Supplementary Figure SF3. Overall, epoch-level accuracy increased for all classifiers compared with the standard DA procedure. A one-way repeated-measures ANOVA revealed the same main effects of preprocessing and base model as previously reported ($F_{2,46}=508.36,p<0.001$ and $F_{2,46}=113.17,p<0.001$ , respectively). Post hoc analyses showed that participant-specific preprocessing still yielded the highest epoch-level accuracy, followed by the combined approach, and then the small preprocessing condition (0.826, 0.819, and 0.733, respectively). Regarding the base models, the overall pattern of results remained consistent with those described in the previous paragraph. PS-GREEN remained the top-performing model, although the performance differences relative to the other models were reduced compared with those observed in the DA procedure. We observed a significant improvement (p<0.001 ) of the pre-trained PS-GREEN strategy as compared with DA, with an increase of the average accuracy of 1.2% (0.825 and 0.813, respectively)

The training time for the fine-tuning in this procedure is significantly smaller than in the DA procedure and is at the same order than for the WP procedure. It is normal as in the training batch, the same number of data is present in this procedure and in the WP procedure.

## 4 Discussion

This study inter-user investigates variability in BCI performance with the long-term objective of facilitating deployment beyond the laboratory. We follow a two-step approach: (i) identifying neurophysiological predictors that predict both inter- and intra-participant differences in performance and (ii) integrating those predictors into a transfer-learning pipeline that shortens calibration time while preserving decoding accuracy. All analyses drawn on the 24-participant dry-EEG burst-c-VEP dataset of Dehais, Cabrera Castillos, et al. (2024) combining visually comfortable stimuli together with dry EEG systems provide a suitable test bed for field-ready BCIs.

### 4.1 Neural correlates of c-VEP BCI performance

We identified five neurophysiological predictors as linear predictors of epoch-level accuracy. The strongest correlation was obtained with the mean correlation between target epochs: participants with more self-similar single-trial responses tended to achieve higher epoch- and trial-level accuracies, as trial-level accuracies are directly linked with the epoch-level accuracies. A plausible interpretation is that sustained engagement or attention leads to more consistent neural responses (Jeunet et al., 2016), which, in turn, provide the decoding model with clearer patterns for mapping from EEG to codes. However, the precise physiological basis of this consistency remains unclear. Exploratory analyses of spectral SNR, spatial pattern drift, and epoch-level misclassifications did not provide definitive explanations. Therefore, cognitive factors such as fluctuating attention or fatigue may still play a role.

Peak-to-peak amplitude of the evoked potential was the second most powerful predictor. Larger voltage excursions improve discriminability in linear and deep decoders alike, echoing the amplitude–performance relation seen in burst-c-VEP BCIs (Cabrera Castillos et al., 2023). These results are also in line with other c-VEP work showing a significant correlation between the amplitude of VEP components and c-VEP BCI performance (Thielen, 2025). More studies are required to reproduce and confirm these neurophysiological predictors for c-VEP BCI performance, to better understand the neurophysiological effects in charge within the c-VEP-based BCI.

The PSD across different frequency bands contributed complementary information: α bandpower (8–13 Hz) and θ bandpower (4–8 Hz) scaled positively with accuracy, consistent with evidence that heightened posterior θ accompanies efficient visuocognitive processing, encoding of new information and an increase in performance (Klimesch, 1999; Puma et al., 2018). However, the results on α bandpower are opposite to previous reports showing an inverse trend of visual α bandpower with performance, argued to be caused by an inattentive state of participants (Klimesch, 1999; Thielen, 2025). To account for discrepancies with portions of the literature, we propose two complementary explanations. First, in the present study, α power is quantified in the analysis-pipeline output space (AP space), that is, after the full preprocessing chain, including spatial filtering and spatial alignment. These operations can change the effective spectral content and spatial distribution of the resulting epochs by emphasizing task-relevant components and attenuating non-aligned or non-task-related sources. In contrast, several previous studies quantified α power in sensor space without comparable filtering/alignment, which may partly explain differences in reported α–behavior associations. Second, although increases in α power are sometimes interpreted as reduced alertness (see for review Dehais et al., 2020), a substantial body of attention research (Foxe & Snyder, 2011; Jensen & Mazaheri, 2010) supports an alternative functional account in which α mediates top–down inhibitory control (“gating by inhibition”). In this framework, elevated α in task-irrelevant pathways suppresses processing of irrelevant spatial locations and visual distractors, thereby facilitating selective processing during sustained attention (e.g., Kelly et al., 2006; Rihs et al., 2007; Worden et al., 2000). This interpretation aligns well with our paradigm: multiple flickering stimuli are presented concurrently, and successful performance requires sustained focus on the target while minimizing interference from peripheral distractors. Accordingly, higher α power may reflect more effective suppression of irrelevant input, thereby supporting better BCI performance. By contrast, higher δ power (<4 Hz) predicted poorer performance—paralleling reports that δ increases under heavy cognitive load or drowsiness (Harmony et al., 1996; Kumar & Kumar, 2016).

Together, epoch-correlation, peak-to-peak amplitude, α enhancement, θ enhancement, and δ suppression explained a part of the inter-participant variance. Crucially, all five neurophysiological predictors can be computed online after only a handful of calibration flashes, making them attractive candidates for real-time quality control and adaptive stimulus adjustment (Thielen, 2025). The PSD features can even be computed from any non-task-related data such as any resting-state interval. While many studies on c-VEP-based BCI focus on improving the decoding pipeline or improving the stimulus paradigm, only few studies focus on understanding the neural mechanisms underlying the c-VEP which remain poorly understood. Improving our knowledge on these neural mechanisms will help to improve the performance in c-VEP-based BCI.

### 4.2 Calibration-efficient transfer learning

Building on these neurophysiological predictors, we investigated the influence of the different preprocessing parts, on the machine learning side. Especially, whether a participant-specific preprocessing pipeline—z-score normalization, xDAWN spatial filtering, super-epoch construction, and Riemannian alignment could homogenize feature spaces across users and thereby facilitate transfer learning. Coupled with lightweight decoders, this pipeline proved highly effective. Among its components, xDAWN contributed the largest gains: the only model without spatial filtering performed >15% worse, and even CNNs with a learnable spatial layer benefited when xDAWN was applied offline (PS-CNN > CNN in the DA setting). This is consistent with the previous observations on neurophysiological predictors, that is the peak-to-peak amplitude of evoked potentials is the second best predictor. xDAWN objective is to find a set of optimal spatial filters to enhance the ERP components (Rivet et al., 2009). As burst-c-VEP stimuli elicit an ERP after each flash, xDAWN spatial filters are a key preprocessing step to improve machine learning decoding.

We further found that participant-specific preprocessing outperformed both the minimal (“small”) and pooled (“combined”) variants. The minimal variant omits a key step, that is the spatial filtering, while the pooled variant estimates preprocessing parameters on multi-subject data. This finding completes the observation regarding the most proeminant neurophysiological predictor described in the previous section, that is, the self-similarity of target epochs at a participant level. The participant-specific preprocessing allows to factor out the major source of variations stemming from the inter-subject variability. The results obtained with the pooled (“combined”) variants highlight that the inter-subject variability introduces noise and parameter mismatch during training, degrading DA performance. Future work should include an ablation study to quantify the marginal contribution of each preprocessing step.

The GREEN architecture (Paillard et al., 2025) retained near-optimal WP performance and, more importantly, preserved accuracy after domain adaptation with only two short calibration trials. The different wavelets bands learned in the GREEN-based algorithm could be the principal factor in the preservation of the accuracy between the WP and DA procedures. The different bands allow the model to capture distinct features that may appear in one band for some participants and in another band for others, thereby building a more invariant feature space. While fine-tuned shallow EEG2Code-derived CNNs and a tangent-space LDA (TS-LDA) pipeline achieved comparable gains, GREEN delivered the best trade-off: the shortest training time (<10s ) and threefold faster inference than TS-LDA—together explaining why PS-GREEN achieved the strongest DA results.

The DA procedure leads to longer training times because the training set is substantially larger than in the WP procedure (approximately 8.5 times larger). However, when classifiers are pre-trained and subsequently fine-tuned on the target participant, training times are reduced and become comparable with those observed in the WP procedure. In this setting, epoch-level accuracy either increases or remains comparable for all classifiers relative to WP. Classifiers using participant-specific preprocessing achieve the best performance, with PS-GREEN and PS-CNN yielding the highest accuracies, further confirming the importance of participant-specific preprocessing. Although the performance gap between PS-GREEN and the other classifiers is reduced under the pre-training strategy, PS-GREEN remains the top-performing model, confirming that it offers the best trade-off between performance and computational cost. PS-GREEN achieved better results with the pre-trained strategy than with DA. However, Supplementary Figure SF6 shows that DA performed best for 20% of the samples, while WP performed best for 15%, suggesting that performance depends on the choice of source subjects and motivating further work on selecting the most suitable sources for a given target participant. Moreover, the reduced training time strengthens the feasibility of using this classifier in online applications.

### 4.3 Limitations

The study is constrained by the fixed two-trial calibration length and is validated on only one single paradigm, dry-EEG dataset. Varying the number and length of calibration flashes will help determine the minimal data required for reliable neurophysiological predictors estimation and alignment. As we can see in Supplementary Figure SF5, the performance increases with the number of calibration size, but it is reaching a sort of plateau. Moreover, the training time increases too. To summarize, a complete study should be performed to find the good trade-off between reducing the training time and the performance. The reported results must be replicated and validated on different independent datasets. Similarly, extending the analysis to wet-electrode or SSVEP/P300 paradigms will test generalizability. Moreover, in this study, we focus for the DA procedure to train with only one source subject. However, the performance of the training could be improved by increasing the number of source subjects or by selecting the best source subjects for the target subject. This could be done by performing multi-source domain adaption or choosing the source subjects with a similar distribution of the target subject. These strategies should be researched in a future study. Another direction would be to test the methods presented in this study within a patient population, as this study focused on healthy users and a patient population may exhibit distinct response characteristics (Verbaarschot et al., 2021).

Although GREEN met real-time timing budgets in this study, fully closed-loop demonstrations are yet to be conducted. However, GREEN has shown a sensibility to preprocessing that could come from the fact that no layer in the model acts as a spatial filter unlike the first two in CNN or the xDAWN layer in TS-LDA.

Future research could pursue at least the following three directions. First, identify the cognitive and physiological drivers of low epoch correlation through concurrent behavioral and peripheral measures. Second, integrate wavelet or multimodal embeddings to exploit cross-frequency and cross-sensor regularities. Third, deploy adaptive calibration schemes that monitor the five markers online and automatically trigger realignment or stimulus reselection when a performance drop is predicted.

### 4.4 Conclusion

By combining interpretable neurophysiological predictors with an alignment-aware transfer-learning pipeline, we demonstrated that dry-EEG burst-c-VEP control can approach high accuracy, without per-user re-training. The inter-epoch correlation, peak-to-peak amplitude, α bandpower, θ bandpower, and δ bandpower neurophysiological predictors provide a principled basis for accounting for inter-participant variability and for on-the-fly performance prediction. The GREEN-based adaptation furnishes a computationally lightweight route to cross-participant generalization.

In practical terms, our results indicate that a user wearing dry electrodes could complete a calibration in under 22 s and achieve decoding accuracies comparable with those obtained after more extensive individual training. Although the present alignment step is performed offline, recent many-to-many and multimodal transfer frameworks (Altindiş et al., 2024; Ding et al., 2025; Sung et al., 2024) may shorten this step further, potentially enabling real-time adaptation on the headset or an edge device. Together, these advances move BCIs a tangible step closer to seamless, out-of-the-laboratory deployment.

## Supplementary Material

Supplementary Material

## Data Availability

Data of the participant are available in zenodo (Dehais, Cabrera Castillos, et al., 2024). Experiment data and code are available on github with the link https://github.com/sebVelut/Predicting-and-overcoming-inter-participant-variability-in-c-VEP-based-BCI.git. A README is available to explain how to launch the scripts.
