# Supplementary material for: Neurophysiological screening of individual variability for robust decoding in c-VEP-based BCI
Source: Imaging Neurosci (Camb). 2026 Mar 20;4:IMAG.a.1172. doi: 10.1162/IMAG.a.1172 (PMC13007385; doi:10.1162/IMAG.a.1172)
Supplement: Supplementary Material [file IMAG.a.1172_supp.pdf]

1 Glossary

c-VEP : code-modulated visual evoked potential

BCI : brain computer interface

EEG : electroencephalography

ERP : event-related potential

SSVEP : steady-state visual evoked potential

MI : motor imagery

CNN : convolutional neural network

LDA : linear discriminant analysis

SNR : signal-to-noise ratio

BP : before preprocessing

AP : after preprocessing

BPWave : before preprocessing and transformed in the wavelet space

APWave : after preprocessing and transformed in the wavelet space

PSD : power spectral density

TS-LDA : tangent space linear discriminant analysis

GREEN : Gabor Riemannian EEGNet

PS : participant specific (for the preprocessing)

C : combined (for the preprocessing)

DA : domain adaptation

WP : within participant

PtDA : pretrained domain adaptation

OLS : ordinary least squares

SMD : standardized mean difference

	DA							WP						
**Index**	**C-GREEN**	**GREEN**	**PS-GREEN**	**TS-LDA**	**CNN**	**C-CNN**	**PS-CNN**	**C-GREEN**	**GREEN**	**PS-GREEN**	**TS-LDA**	**CNN**	**C-CNN**	**PS-CNN**
**P1**	0.731	0.587	**0.94**	0.713	0.787	0.765	0.912	0.913	0.541	0.894	0.903	0.903	**0.921**	0.919
**P2**	0.724	0.573	**0.849**	0.727	0.819	0.784	0.81	0.793	0.542	0.804	0.78	0.834	**0.835**	0.797
**P3**	0.694	0.568	**0.819**	0.688	0.75	0.719	0.77	0.778	0.536	0.772	0.796	**0.804**	0.796	**0.804**
**P4**	0.782	0.588	**0.912**	0.783	0.818	0.806	0.886	0.853	0.529	0.87	0.842	0.834	0.881	**0.883**
**P5**	0.697	0.561	**0.849**	0.67	0.773	0.731	0.821	0.769	0.511	0.827	0.83	0.816	**0.854**	0.834
**P6**	0.79	0.584	**0.891**	0.759	0.839	0.837	0.863	0.872	0.545	0.867	0.831	**0.899**	0.874	0.88
**P7**	0.725	0.552	**0.858**	0.724	0.788	0.758	0.819	0.788	0.524	0.81	0.788	**0.83**	**0.83**	0.825
**P8**	0.793	0.636	**0.904**	0.767	0.854	0.818	0.864	0.888	0.597	0.848	0.877	0.844	**0.891**	0.857
**P9**	0.677	0.55	**0.776**	0.646	0.71	0.681	0.743	0.758	0.506	0.753	0.705	0.752	**0.761**	0.756
**P10**	0.586	0.525	**0.745**	0.598	0.607	0.601	0.69	0.678	0.495	0.644	0.584	0.563	**0.698**	0.675
**P11**	0.711	0.547	**0.869**	0.686	0.77	0.762	0.808	0.852	0.513	0.823	0.825	0.818	0.852	**0.855**
**P12**	0.711	0.577	**0.825**	0.677	0.753	0.726	0.78	**0.802**	0.566	0.775	0.789	0.773	0.787	0.786
**P13**	0.725	0.574	**0.815**	0.712	0.776	0.761	0.811	0.800	0.561	0.809	0.739	0.788	0.82	**0.837**
**P14**	0.749	0.579	**0.828**	0.743	0.783	0.774	0.8	0.761	0.529	0.84	0.822	0.792	**0.85**	0.846
**P15**	0.584	0.524	**0.749**	0.61	0.645	0.622	0.694	0.609	0.518	0.67	0.679	0.683	0.697	**0.719**
**P16**	0.749	0.6	**0.892**	0.734	0.81	0.773	0.857	0.835	0.573	**0.864**	0.805	0.787	0.837	0.836
**P17**	0.728	0.582	**0.94**	0.713	0.790	0.763	0.900	0.882	0.566	0.913	0.913	0.881	0.919	**0.921**
**P18**	0.772	0.585	**0.868**	0.701	0.779	0.783	0.781	0.818	0.543	0.816	0.728	0.789	0.814	**0.837**
**P19**	0.649	0.531	**0.77**	0.604	0.697	0.695	0.759	0.701	0.504	0.754	0.747	**0.767**	0.755	0.756
**P20**	0.7	0.553	**0.816**	0.689	0.769	0.744	0.787	0.771	0.515	0.776	0.726	0.771	**0.812**	0.791
**P21**	0.757	0.657	**0.935**	0.724	0.816	0.799	0.889	0.890	0.601	**0.909**	0.9	0.88	0.889	0.891
**P22**	0.685	0.57	**0.834**	0.655	0.715	0.722	0.793	**0.810**	0.507	0.74	0.728	0.747	0.78	0.805
**P23**	0.646	0.557	**0.741**	0.63	0.688	0.676	0.675	0.705	0.523	0.688	0.72	0.727	**0.737**	0.733
**P24**	0.826	0.636	**0.94**	0.781	0.878	0.844	0.913	0.863	0.578	0.906	0.873	0.897	0.913	**0.914**

Table 1: ST 1. Balanced epoch-level accuracy calculated for all participants and all decoding model in WP and DA training procedure. For both training procedure and each subject, the best results are written in bold. PS-GREEN is always the best decoding model in DA training procedure, whereas the best decoding model is varying between the CNN-based model in WP training procedure.

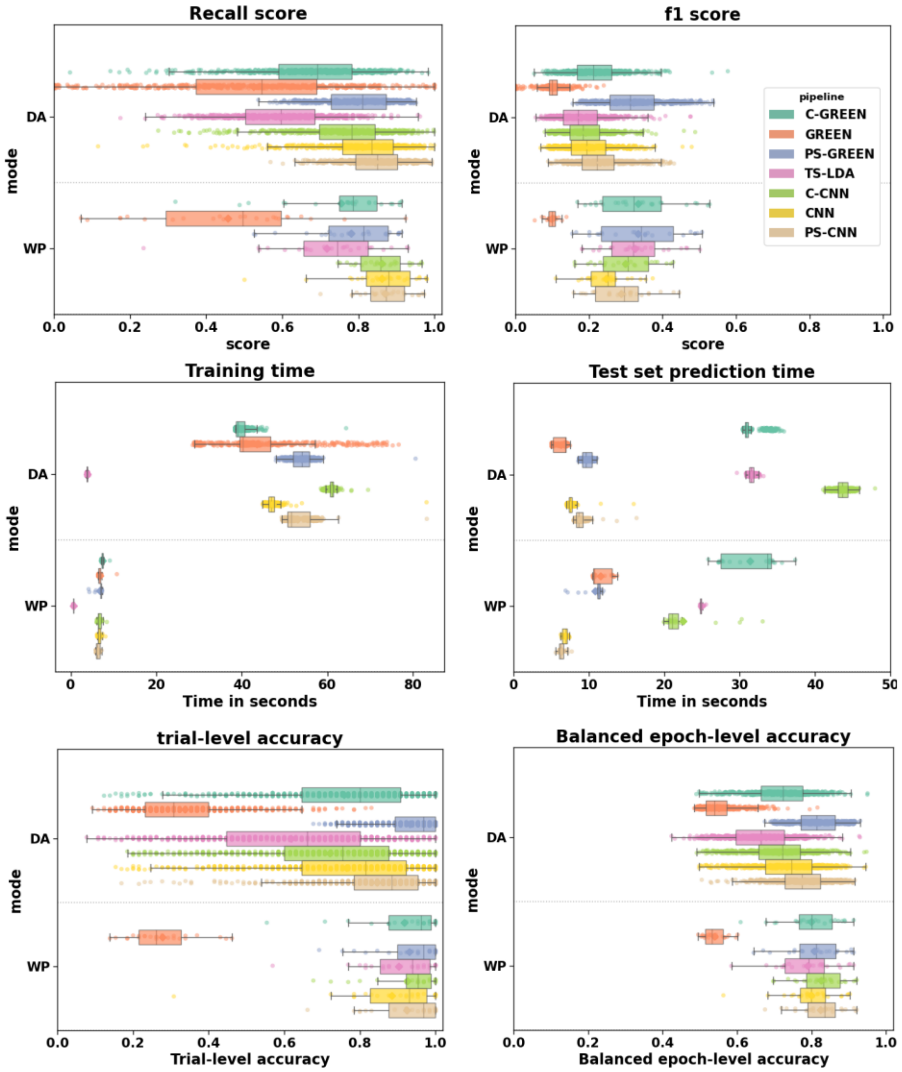

Figure 1: SF 1. The 6 measures of performance - trial-level and epoch-level accuracy, f1 and recall score, training and prediction time - of the different decoding pipelines described in this paper. On each graph, the upper half is the measures of the DA training methods, the lower half is for the measures of the WP. The boxes correspond to the interquartile. The line in the middle corresponds to the median. The diamond-shape point corresponds to the mean.

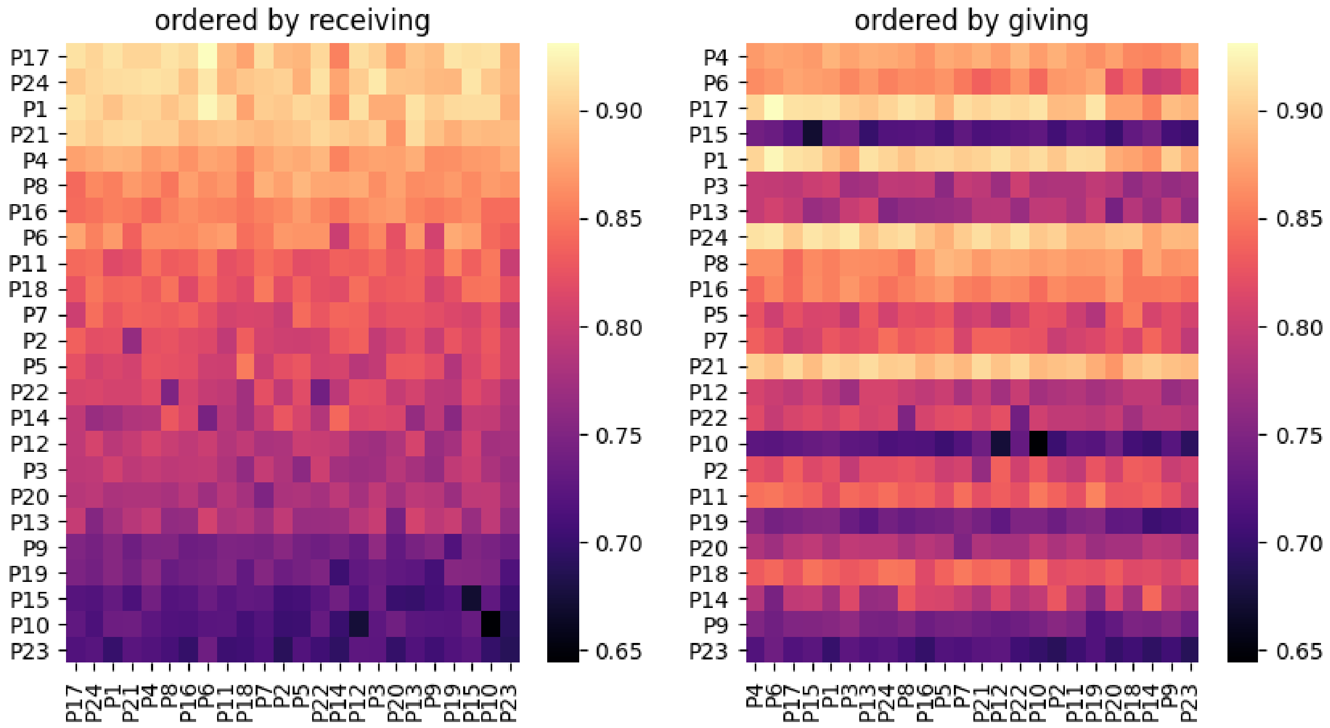

Figure 2: SF 2. Transfer matrices between each participant. The left ones are sorted along the sum of the trial level accuracy of the concern participant trained once with each other participant (along the performance in receiving information). The right ones are sorted along the sum of the trial-level accuracy of each other participant trained with the concern participant (along with the performance in transferring information). The trial-level accuracies shown here are the ones obtained with the decoding model PS-GREEN. We can see an apparent order for the "receiver" but not for the "giver".

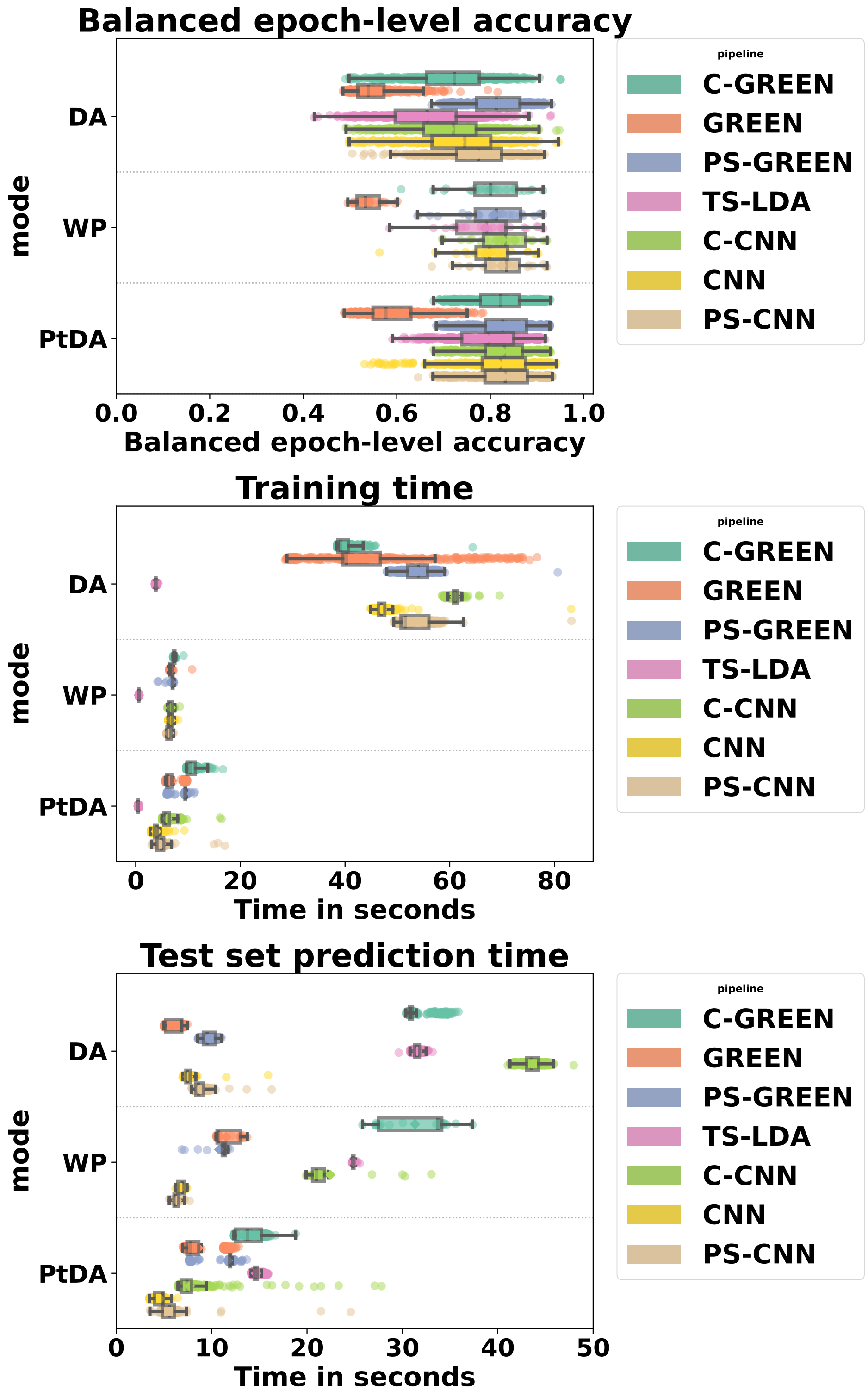

Figure 3: SF 3. Epoch-level accuracy, training time and prediction time of the different decoding pipelines described in this paper. The pretrained domain adaptation (PtDA) procedure has been added in each graph on the lower part compared to the figure 2.4 of the paper. We can see an overall better epoch level accuracy in this PtDA procedure compare to DA. However we can perceive a much smaller training time.

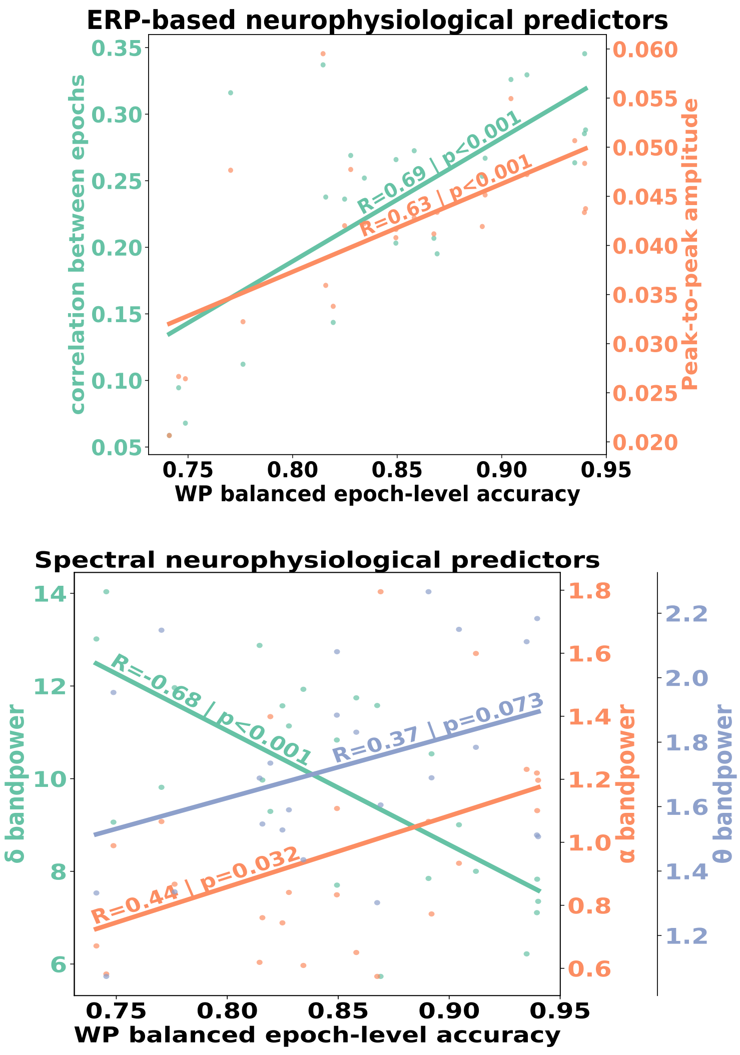

Figure 4: SF 4. Spectral and ERP predictors (α, θ and δ bandpower, peak-to-peak amplitude and inter-epoch correlation) for PTGREEN classifier in AP measurement space within DA procedure. The results are the same than within WP procedure except for the θ bandpower which became non-significant.

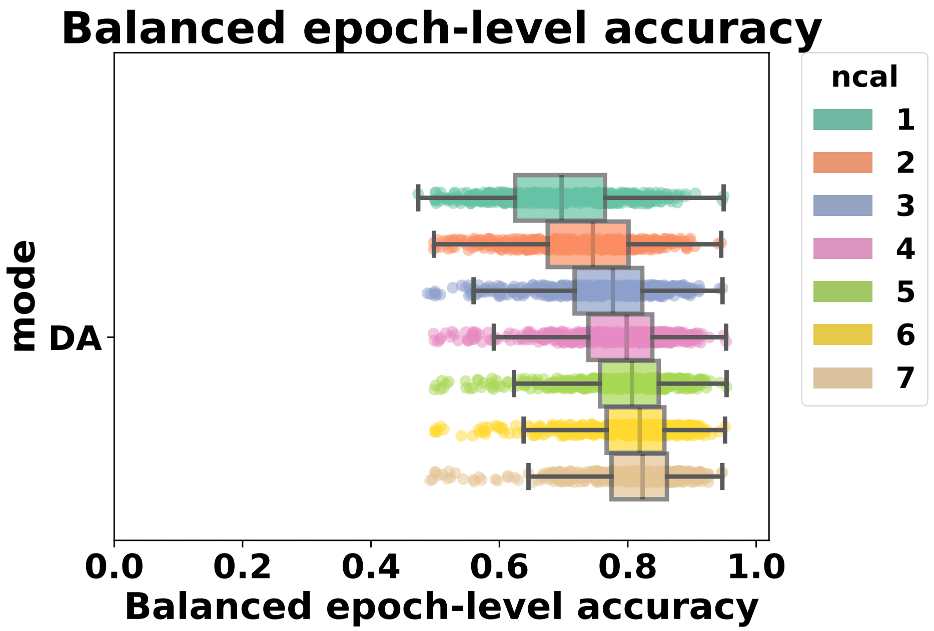

Figure 5: SF 5. Epoch-level accuracy depending on the number of runs, on the 15 present per participant, used in the training batch, ie the calibration set.

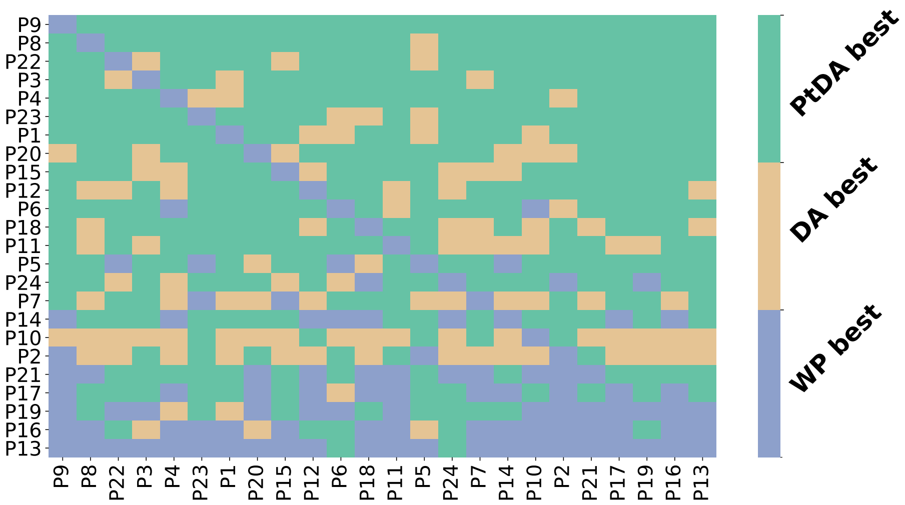

Figure 6: SF 6. Comparison between the performance with pre-trained domain adaptation (PtDA) strategy and the performance with domain adaptation (DA) strategy and the within participant strategy (WP). The gray square corresponds to a case where WP is the best strategy. A light brown square corresponds to a case where DA performs the best. A light green square corresponds to a case where PtDA is the best strategy.
